# Marker-Based Movement Analysis of Human Body Parts in Therapeutic Procedure

**DOI:** 10.3390/s20113312

**Published:** 2020-06-10

**Authors:** Muhammad Hassan Khan, Martin Zöller, Muhammad Shahid Farid, Marcin Grzegorzek

**Affiliations:** 1 Research Group for Pattern Recognition, University of Siegen, Hölderlinstr 3, 57076 Siegen, Germany; developer@janunn.de; 2 Punjab University College of Information Technology, University of the Punjab, Lahore 54000, Pakistan; shahid@pucit.edu.pk; 3 Institute of Medical Informatics, University of Lübeck, Ratzeburger Allee 160, 23538 Lübeck, Germany; grzegorzek@imi.uni-luebeck.de

**Keywords:** movement analysis, angle orientation, physiotherapy, rehabilitation

## Abstract

Movement analysis of human body parts is momentous in several applications including clinical diagnosis and rehabilitation programs. The objective of this research is to present a low-cost 3D visual tracking system to analyze the movement of various body parts during therapeutic procedures. Specifically, a marker based motion tracking system is proposed in this paper to capture the movement information in home-based rehabilitation. Different color markers are attached to the desired joints’ locations and they are detected and tracked in the video to encode their motion information. The availability of this motion information of different body parts during the therapy can be exploited to achieve more accurate results with better clinical insight, which in turn can help improve the therapeutic decision making. The proposed framework is an automated and inexpensive motion tracking system with execution speed close to real time. The performance of the proposed method is evaluated on a dataset of 10 patients using two challenging matrices that measure the average accuracy by estimating the joints’ locations and rotations. The experimental evaluation and its comparison with the existing state-of-the-art techniques reveals the efficiency of the proposed method.

## 1. Introduction

Motor disabilities are the partial or total loss of the body part’s functionality due to damage in the central nervous system, which controls the body movement. Neurological physiotherapy aims at restoring the patient’s ability to perform his/her daily life activities independently by repairing the central nervous system. To this end, the Vojta techniques [1,2] and the neurodevelopmental treatment [3,4] are the most common approaches used by the physiotherapists to deal with the motor disabilities in patients. The Vojta techniques are based on the principle of reflex locomotion [1], which enables the elementary patterns of movement in patients by stimulating the appropriate reflex points on the patient’s body region. The neurodevelopmental treatment tends to restore the patient’s normal movements and inhibit the abnormal movements through positioning and handling techniques such as giving a massage, exercises of lying in a prone position, and stimulating the key points of motion. A neurological physiotherapy is a widely recommended treatment to deal with the structural disorders of muscles and joints. In particular, they are found to be very useful in dealing with cerebral palsy, hip joint dysplasia, disturbance in central coordination, etc. [5,6].

Upon diagnosing the motor disabilities, the doctors or physiotherapists design a therapy session of 5–20 min for the patient. It is often performed several times in a day or week and determined in regular intervals based on the patient’s development. It may be continued for several weeks based on the patient’s recovery. Due to limited staff at hospitals and prolonged time period for rehabilitation, an in-home continuation of the therapy is very helpful. Moreover, the frequent visits to a therapist’s clinic make the treatment expensive too. Therefore, the therapist may suggest in-home therapy. The therapist explains the objectives and methodology of the treatment to the patient’s caretaker. However, an inaccurate therapy may either reduce the effectiveness of the treatment or even be harmful for the patient [7]. Therefore, a visual tracking system is needed for motor rehabilitation to track the motion of the body parts during the therapy program to validate whether they are being performed correctly or not. Moreover, this movement analysis can only be validated by a well-trained expert clinician and they are not widespread, especially in poor countries. Hence, such a system would be very useful to reduce the need of experts and the acquired motion information of body parts may help the patient to observe the rehabilitation over time by providing an accurate evaluation of an in-home therapy.

Numerous vision-based techniques for clinical human motion analysis have been proposed [8,9,10,11]. Some are marker-based motion tracking methods, for example, [12,13,14,15]. They use markers on the human body region to represent the joints’ locations and used them to detect and track skeletal information. These methods, however, require expensive equipment or they are limited to two-dimensional motion information, which makes them impracticable for in-home therapy. Some techniques, for example, [10,16,17,18,19], use various image features to track the human body parts; they do not use any markers. Markerless techniques need only conventional cameras instead of special cameras and intrusive markers; however, they are computationally expensive and their accuracy may degrade drastically when the desired body parts are occluded [10]. Other techniques, such as [20,21,22], use the integrated skeleton information of human from Microsoft Kinect to encode the motion information. Although, the Kinect sensor provides real-time human skeleton tracking but imposes restrictions on the body size and standing positions [23,24]. The techniques such as [25,26,27] use different sensors for body movement analysis.

This paper presents a computer-aided system that allows three-dimensional (3D) acquisition of unconstrained movement in patients during the therapy process. Here and in the rest of the text, 3D represents the color and its corresponding depth information. It uses Microsoft Kinect sensor, which is far less intrusive and a lot cheaper than existing commercial solutions. The motion information at various body parts of the patient is computed and their distinct motion patterns are analyzed to evaluate the therapeutic procedure. In particular, the proposed method begins by detecting different body parts using color markers attached to joint locations. Various features are computed to validate the marker objects. The estimated joint locations are tracked in the video stream and angles are computed at predicted joints such as knee, elbow, shoulder. The motion information using angle orientations at different joints in the temporal direction is computed and encoded, which is employed to validate the accurate movement patterns. It is concluded in our earlier research [10] that the movements computed in two-dimensional (2D) space do not provide sufficient information, therefore the proposed method exploits the 3D position of human body parts to encode the desired movement using Microsoft Kinect sensor. In order to validate the performance of the proposed system, a dataset of 10 patients is collected in a local hospital. The patients were suffering with motor disabilities and they were being treated by the therapists in the recording days. The performance of the proposed method is evaluated using two challenging matrices and the results are compared with existing state-of-the-art. The results showed that the proposed system is effective and efficient. Moreover, it comprises only a few standard components and can be manufactured easily in a limited budget for in-home rehabilitation programs.

## 2. Background

**Figure 1:**
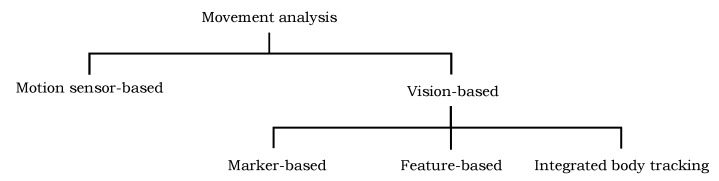
Categorization of existing techniques for movement analysis of human body parts into different groups.

The motion information of different body parts of a patient is important for better clinical and behavioral assessments and efficient therapeutic decisions. In recent years, numerous automated and semi-automated techniques have been proposed to support physiotherapy and rehabilitation programs to restore the functional ability of the patients; they can be categorized into two groups: vision-based approaches and motion sensor-based approaches (Figure 1). The short description of a few techniques in each group is presented in the following.

### 2.1. Vision-Based Algorithms

The vision-based approaches can be further distributed into three groups based on the underlying motion capturing model. The first family of techniques make use of markers on the human body region to represent the joints’ locations and use them to detect and track their information in the video. The second group of techniques employ the image features, such as shape, color, and edges, to detect and track the human body parts in video and encode their motion information. The techniques in the third group are also marker-less; they estimate the joints’ locations using the integrated body tracking functionality of the Kinect sensor.

#### 2.1.1. Marker-Based Techniques

The marker-based techniques capture the motion information based on the tracking of markers, for example, reflective spheres, light-emitting diodes, or infrared markers. The markers are attached on the target regions, such as limbs, and their position and orientation are tracked. The recognition of marker based object features in the video and their displacement information over time is used to record the continuous time-series data, which represents the dynamic limbs movement. For example, the research in [13] proposed to attach different color markers on joints’ locations and they are tracked in successive frames of the video. Similarly, two games are proposed in [12] for upper limb stroke rehabilitation. The authors employed color objects that are attached to the upper limbs; the algorithm detects them using a calibration process and tracks them in the video to encode the motion information.

Rado et al. [15] proposed an unsupervised patient rehabilitation method using marker based motion tracking of knee movements with the help of an infrared optical tracker. The errors are detected in the desired movement and demonstrated to the user how to perform the movement correctly. The research in [14] introduced a motion analysis system using seven visual cameras to capture the 3D motion information of markers attached to a human body region. The motion information is used in predicting the risk for developing movement disorders. Paolini et al. [28] proposed a rehabilitation system for gait training using the tracking of foot positioning and their orientation with the help of color markers. The authors in [29] proposed a 3D position sensing gadget using a tiny high resolution video camera and a fixed infrared emitting target to encode the human motion information. A therapy system for upper limb rehabilitation is presented in [30]. A recent survey on the evaluation of the marker-based system is presented in [31].

Several commercial optoelectronic systems have also been proposed to capture human movement. The majority of such systems employ a setup of multiple cameras that emit invisible infrared light and passive markers that are placed on the human body and reflect this infrared back to the cameras allowing to estimate their 3D position. Studies on widely used commercial marker-based systems and vision-based human motion capturing systems are presented in [32,33,34].

Most of the marker-based techniques are either dependent on the installation and calibration of multiple cameras or limited to encode two-dimensional motion information. Moreover, the use of infrared based marker sensors and multiple cameras requires extra equipment, hence they are quite expensive and may not be suitable for home-based rehabilitation.

#### 2.1.2. Feature-Based Techniques

Feature-based techniques are markerless solutions to detect and track the human body parts. They use image features, such as shape, color, edges, and pixel coordinates, to detect body parts and track them in the video to encode their motion information. For example, the techniques proposed in [16,35] applied the body part model fitting technique, i.e., shape matching, on depth images to segment the patient’s body region. Later, several features are computed from the segmented shape to capture their movements. Similarly, the authors in [18] proposed a model fitting technique into the depth image of a Kinect sensor, targeting rehabilitation exercises during physiotherapy. They record the motion information of a human leg by fitting a model. The method proposed in [36] recovers the full head motion from an input video using a head template-model. The algorithm proposed in [10] applied a deformable part-based modeling technique to detect the body parts of the patient in images. The detected locations are tracked in the subsequent frames of the video to encode the motion information. The authors in [17,37] exploit the color features and pixel locations to segment the patient body region from the images, and proposed several statistical measurements and geometrical features on the segmented region to encode the movement.

Shotton et al. [38] proposed a method to estimate the 3D position of joints in the depth image. They applied the depth comparison features for each pixel and random forest classifier is used to classify them into different body parts. Hesse et al. [39] improved the method in [38]. Rather than using a random forest classifier, they employed random ferns for pixel-wise body part classification to estimate the infant’s body pose. Further, they computed angle orientation at predicted joints to encode their respective motion information. Evett et al. [40] developed a game for stroke rehabilitation using the movements and gestures of hands. Several techniques such as [41,42,43] investigated the optical flow information of human body-parts to analyze the movement patterns. However, they suffer with the localization of movements at particular joints. Feature-based techniques do not require any intrusive markers on the human body region and seem to be attractive as most of them only need a conventional camera instead of special cameras. However, most of these techniques are computationally expensive and their accuracy may degrade drastically when the desired body parts are partially occluded [10].

#### 2.1.3. Integrated Body Tracking Functionality

Recently, the Microsoft Kinect sensor has emerged as an effective and economical gadget in clinical investigation and rehabilitation places to provide the movement analysis of human body parts [10]. It comprises of a visual and a depth sensor, which provides color and depth information of the captured scene, respectively, and helps to construct a 3D view of the environment. Furthermore, the depth sensor of the Kinect provides skeleton information of the human and its tracking in the video can be used to encode the respective motion information of the joints. It has been used by several researchers at ambient assisted living, therapeutic and rehabilitation places to analyze their movemen patterns.

Exell et al. [21] used the integrated skeletal tracking information of the patient from the Kinect sensor to analyze the rehabilitation in upper limbs. Similarly, in [22], the skeletal information from Kinect is used to develop a system to compare and evaluate the patient’s movement. A rehabilitation system is developed in [20] to assist the therapists in their work. It is designed for the patients suffering from motor disabilities and presents the rehabilitation progress to the therapists. Wu et al. [44] computed the 3D coordinate distances between 15 human joints using the integrated skeletal information of Kinect, and used them to monitor the rehabilitation progress. In [45], a system was proposed to validate the poses of human during the therapy exercises. It uses the patient’s posture knowledge from Kinect integrated skeletal information and validates it with the model posture. The techniques proposed in [46,47] developed the rehabilitation systems for the patients suffering from motor disabilities using the Kinect skeleton tracking. Chang et al. [48] proposed a motion tracking system for the rehabilitation of upper limbs using Kinect. The usage of Kinect at different therapeutic and rehabilitation places is reviewed in [49,50].

The invention of a low-cost 3D Kinect sensor and its integrated body tracking abilities have triggered a significant amount of research on human motion analysis, clinical assessment, and rehabilitation. However, the limitation in which the size of the subject being integrated for body-tracking should be greater than one meter and should be in an up-right position in front of the sensor prevents the automatic detection and movement analysis of patients, in particular, children. Moreover, the skeletal information of the patient cannot be extracted if some body parts are occluded, which is common during the therapeutic procedure.

### 2.2. Motion Sensor-Based Algorithms

Recently, numerous researchers have exploited the Inertial Measurement Unit (IMU) to encode human movement. An IMU is the combination of accelerometers, gyroscopes and magnetometers, which encode the motion information relevant to acceleration in the sensor/body, angular velocity and magnetic field around it, respectively [51]. The accelerometer and IMU are the frequently used sensor technologies in encoding the motion information by estimating the limb kinematics and/or trunk posture [52]. For example, the technique presented in [53] employed IMUs to assess the post-traumatic rehabilitation. They derived the motion trajectory of the whole movement using IMU’s to track basic exercises related to specific planes of movement and/or rotation. The movements of the patient were graphically represented to obtain immediate visual feedback by comparing it with the reference movement. Luo et al. [54] presented an interactive virtual reality system for arm and hand rehabilitation. They used two IMU’s along with one Optical Linear Encoder (OLE) to encode the arm motion. The authors in [25] employed a set of IMU’s and smart shoes with pressure sensors to analyze the gait in rehabilitation process. They exploited the information of pressure sensors to estimate the force distributions between the two feet during the walk. Similarly, the authors in [26,55] used a set of IMU’s to detect body movement and estimate the joints motion information. In [56], the researchers attached four accelerometers with the upper and lower limbs, and use their data to encode the respective motion information. Later, they employed a decision tree algorithm to distinguish these movements into healthy and abnormal. Chen et al. [57] presented a method to assess the rehabilitation progress of a patient suffering with knee osteoarthritis. They employed tri-axial accelerometers on the chest, thigh and shank of the working leg to encode their motion information. Several techniques have been proposed in the literature that employed a set of accelerometers to encode the human motion information for a home-based rehabilitation system [58] and to monitor the patient movement [59,60]. A few studies, such as [61,62], have also proposed where the IMU sensors are integrated in a wearable garment rather than attaching to the human body.

Since such techniques require multiple sensors on the human body to capture the motion information at different joints, besides the financial aspects, they require their cumbersome installation and calibration. The adaptation of such a configuration may be more complex when the therapy is given at home in the absence of a technical person. Wearable sensors cannot be rigidly fixed to the bone and a motion artifact may have occurred [63]. They may produce potential effects in the data analysis due to the noise of sensor motion [64]. Moreover, wearing a number of sensors on the human body may cause discomfort to the patients (particularly for young patients and babies) and may affect their natural movements [17].

## 3. Data Acquisition

**Figure 2:**
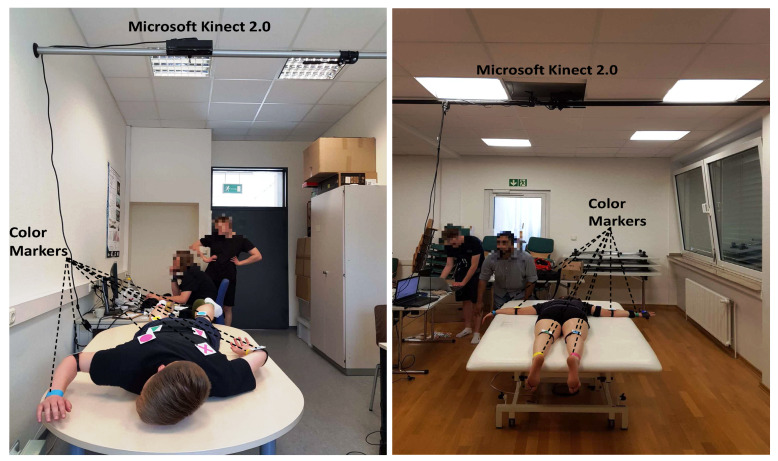
Camera setup during the recording in the lab (**left**) and in the hospital (**right**). The camera (Microsoft Kinect 2.0) was hooked with the help of an autopole at the height of 2 m with an angle of 90° from the table surface where the patient is lying for therapy.

To the best of our knowledge, no public marker-based dataset is available to analyze the movement of the patients during the physiotherapy, therefore we captured a dataset in a local hospital [65] using Microsoft Kinect 2.0, which was hooked with an autopole at the height of 2 m with an angle of 90° from the table surface, as shown in Figure 2. The setting was chosen to be in accordance with the recommendation for capturing the best data quality, clear visibility of movement patterns with minimum occlusion, during the treatment. In the initial phase, test video sequences were captured in our lab [66], and later, the same configuration was adopted at the hospital for the data capturing of real patients. A dataset from 10 patients of both genders having movement disorders is collected. Based on their availability and frequency of treatment in the hospital by the therapists, 8 to 20 recordings were made for each proband, generating a total of 165 videos.

During the recording, both color and depth frames are captured, and they are temporally synchronized. The obtained color images have the size of 1920 × 1080, whereas the depth images have dimensions of 512 × 424. We validate the timestamps for each of the color and depth frames. It is particularly observed from the behavior of frames’ timestamps and sequence numbers that there is always a delay of a few milliseconds between the data of these two sensors, the lowest possible value being 6.25 milliseconds (ms). The experiments revealed that this delay is not observable when looking at the frames and therefore negligible. However, our algorithm drops the frames on the maximum difference of 6.25 ms to reliably compute the 3D information. In this way, the proposed framework is able to capture both the color and the depth streams at the rate of 25 frames-per-second (fps).

## 4. Proposed Movement Analysis Method

**Figure 3:**
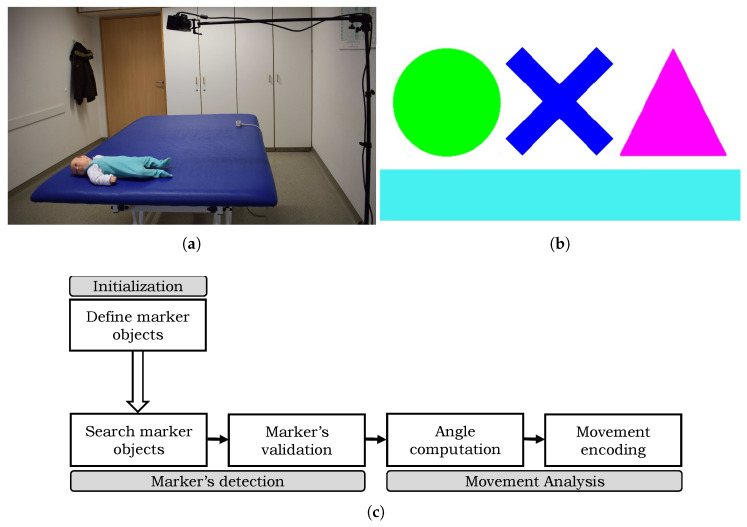
Proposed method (**a**) an illustration of the hardware system components, (**b**) selected color markers with predefined geometric shapes of circle, cross, triangle and bar, and (**c**) block diagram of the proposed system.

The proposed movement analysis framework works in two steps. In the first step, detection and tracking of the markers in consecutive video frames commences. For each successive color frame, the algorithm looks for the marker in the vicinity of its position in the preceding frame. During therapy, a marker may get occluded by the therapist or other objects resulting in detection failure. In such cases, the marker position is approximated using a probabilistic estimation approach. In the second step, the joint coordinates are estimated at detected locations. The proposed method computes angle orientations at detected joint locations and they are tracked across the video to encode the respective movement. A block diagram of the proposed method is shown in Figure 3c. The detail of each step is described in the following sections.

### 4.1. Initialization

The proposed movement analysis framework exploits color markers with predefined geometrical shapes and colors. Magenta, red, green, yellow, blue and cyan colors are chosen because they have distinguishable hue values in the Hue Saturation Value (HSV) that helps in better tracking. A white border around these shapes makes sure that they do not overlap with the color of the patient’s clothing, skin, or any other object in the surrounding. The color markers used in the proposed framework are shown in Figure 3b. Initially, these markers are printed on a standard printing paper and attached either to a piece of foam or soft plastic-sheet to make them more durable but still deformable, which is important when marking joints’ locations that may bend towards the direction of the marker during the treatment. The markers are attached on the patient body region either using pins on his/her clothing or using velcro on body parts, such as at the wrist or ankle.

The intensity values of the marker objects are sensitive to illumination changes and recording environment. In order to adjust the color ranges of the marker, in the first frame of the recording their color ranges and locations are saved by clicking on each marker. The proposed algorithm provides a simple slider approach to adjust the range of each hue, saturation, and intensity value until all markers are outlined correctly. The movement of the therapist or the patient during the treatment can easily create shadows, lowering the markers intensity value. Therefore, the low and the high range of each color has to be chosen quite broadly to reliably find the markers throughout the whole video. The slider approach needs to be adjusted only once for a therapy setup, and later the same color ranges can be used for every following session recorded in the same setup settings. The aim of this step is to make the algorithm capable of reacting to changes in the marker’s appearance due to artificial or bad lighting. Moreover, by clicking on the marker, a region of interest (i.e., marker’s selection) is obtained with the coordinates of its center. The proposed algorithm computes various features from the region of interest and a marker object is registered. All marker objects are initialized in this manner. We also evaluated ArUco markers [67] in our experiments and found that our color markers perform better in detecting and tracking different body-parts during the therapy process.

### 4.2. Marker Detection

The marker detection is achieved using its color and shape information. A region of interest (ROI) in the current frame is established based on the marker position in the preceding frame. The contours are detected in the region of interest and algorithm looks for contours within the range of the marker’s known color to avoid false positives. The resulting set of contours may contain outliers if the ROI is quite large. To deal with this problem, a double thresholding technique is applied on each candidate area to remove invalid contours. The low and high threshold ranges on the contour area were determined empirically. The proposed algorithm iterates over the vector of candidates, calculates their area and chooses to keep or remove it based on the threshold ranges. This step helps to eliminate most of the false positives, and those that survived can be eliminated by adopting a suitable similarity measure.

The proposed algorithm computes different features from the contour area to appropriately describe the two-dimensional geometrical shape of the marker objects. The features we use are area, contour convexity, estimated object center position, and object color. The features are combined to obtain a feature vector, which is used in the detection. Rather than using a simple binary convexity measure, the convexity of the contour is defined as the ratio of contour area and its convex hull area:

(1) $$Convex=\frac{A}{C}$$

where *A* and *C* represent the area of the contour and the area of the convex hull of the contour, respectively. The value of Convex describes how much the convex hull of the contour is filled. The position of the marker is approximated as the center of the contour’s bounding box. This yields coordinate points on the horizontal and the vertical axis in color space. Since Microsoft Kinect is being used, these coordinate points can be used to get their corresponding depth values using the camera extrinsic and intrinsic parameters to obtain the 3D information of the scene. The resulting 3D coordinates are then used for angle computation to encode the movement (Section 4.3). The computed features from all the candidate marker objects are compared with the actual marker features for their validation. The distance between two features is calculated using Euclidean distance $d_{L_{2}}$, resulting in three values that are accumulated to get the overall distance between the actual marker object and each of the marker candidate.

(2) $$d_{L_{2}}:R\times R\to R_{0}^{+},d_{L_{2}}\left(x_{1},x_{2}\right)=\sqrt{{\left(x_{1}-x_{2}\right)}^{2}}$$

where $x_{1}$ and $x_{2}$ represent the respective feature values of the candidate and the actual marker. However, Equation (2) may provide different ranges in feature comparison, for example, the area distance is significantly higher than the convexity distance, which makes it difficult to analyze the data. To solve this problem, *z-score normalization* [68] is used. It defines the number of standard deviations between a data point and the average. The mean (μ) and standard deviation (σ) of each feature is computed:

(3) $$\mu =\frac{1}{n}\sum_{i=0}^{n-1}x_{i}$$

where *n* is the total number of frames that are processed, and $x_{i}$ is the value of a feature in frame *i*.

(4) $$\sigma =\sqrt{\frac{1}{n-1}\sum_{i=0}^{n-1}{\left(x_{i}-\mu \right)}^{2}}$$

The z-score of a feature *x* is calculated as,

(5) $$z=\frac{x-\mu }{\sigma }$$

Assuming a Gaussian distribution, it is plausible to expect z∈[0,3] for marker candidates similar to the actual marker; and z>3 for the candidates that are most likely not a marker object. Finally, the distance between a marker and its candidate $d_{z}$ is the accumulated distance of the normalized feature values,

(6) $$d_{z}=0.4\times z_{area}+0.3\times z_{convex}+0.15\times z_{x}+0.15\times z_{y}$$

where $z_{area}$, $z_{convex}$, $z_{x}$, and $z_{y}$ are the normalized area, convexity, and the *x* and the *y* coordinates of the estimated center position, respectively. The proposed distance function employs weights to define the influence of each feature. The value of these weight factors are selected empirically. Initially, all the parameters are initialized with equal factors of weights, for example, 25% weight is assigned to each of the parameters. However, their values are tuned to get the optimal detection results.

The pooled distance of Equation (6) is converted into a similarity value $sim_{z}$ that falls in [0,1]. It is easier to define a threshold if the maximum similarity value is known, opposed to a distance value. Therefore, a similarity function is needed such that the distance 0 should be converted to the maximum similarity and the similarity should be minimal if the distance is maximum. We recall that an object with a z-score greater than 3 is most likely not the marker object. We defined the conversion function as:

(7) $$sim_{z}=\{\begin{array}{cc} \left(1-\frac{d_{z}}{3}\right) & \mathrm{if}d_{z}<3\\ 0 & \mathrm{otherwise} \end{array}$$

In addition to the color and distance of the marker objects, their shape similarity is also used in detection. The contour shapes are matched using Hu moments [69,70]. The shape distance $d_{s}$ defines the shape similarity in terms of distance between the candidate’s contour and the desired marker’s contour. This distance is then also converted to similarity using the following function:

(8) $$sim_{s}=e^{-d_{s}}$$

The overall similarity sim is computed as the weighted sum of the z-score similarity (Equation (7) and shape similarity (Equation (8)).

(9) $$sim=0.75\times sim_{z}+0.25\times sim_{s}$$

All the candidates markers with similarity less than a threshold value τ are discarded. We use non-maximum suppression, and the contour with the highest similarity (Equation (9)) is greedily picked as an estimation of marker object. However, if the algorithm is unable to detect the marker in the vicinity of its last position, it is executed on the whole image. If this step fails, it means the marker is most likely occluded. To solve this problem, the algorithm approximates its position using a probabilistic estimation approach. We use the Kalman filtering [71] technique, a variant of Bayes filters [72], to estimate the location of the marker object. Knowing the initial position of an object, it is able to predict the most likely position of that object at a given time. It may be noted that this method assumes the system to be a *Markov*, i.e., all the necessary information to predict its next state is given by its current one. Following this assumption, the current estimated state can be used for the next prediction.

### 4.3. Movement Analysis

**Figure 4:**
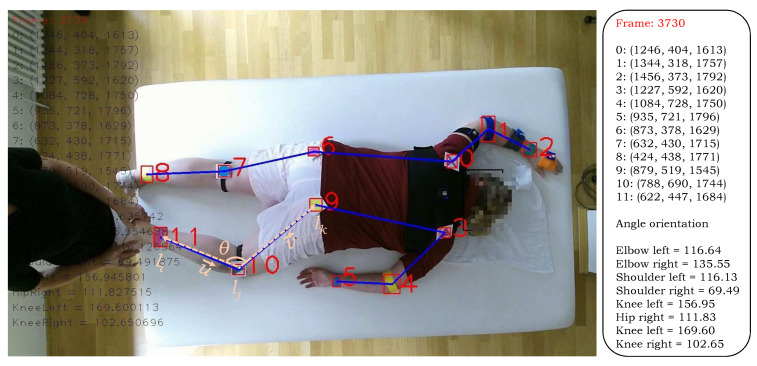
A sample image showing the position of markers on the human body. The markers are labeled from 0 to 11; their 3D position (horizontal-axis, vertical-axis, and its depth), and angle orientations at different joints are annotated at the right side of the image. The way of angle computation at the right knee is also elaborated.

The proposed algorithm computes angle orientation at the estimated locations of the markers and their tracking in the temporal direction is used to instigate the movement in various parts, such as elbow, shoulder, knee. Let us consider the case of elbow connected with the shoulder and wrist. Let $l_{i}$, $l_{j}$ and $l_{k}$ denote the wrist, elbow, and shoulder joints respectively (Figure 4), the following two vectors are computed:

(10) $$\begin{array}{c} \vec{u}=l_{j}-l_{i}\\ \vec{v}=l_{j}-l_{k} \end{array}$$

The angle orientation at the right knee in the sagittal plane is computed using the vectors $\vec{u}$ and $\vec{v}$,

(11) $$\theta \left(\vec{u},\vec{v}\right)=cos^{-1}\;(\frac{\vec{u}\cdot \vec{v}}{\left|\vec{u}\right|\cdot \left|\vec{v}\right|})$$

Since Kinect provides the depth information of the object in the scene along with its color information, these angles are computed in the 3D domain. That is,

(12) $$\begin{array}{c} \vec{u}\cdot \vec{v}=u_{x}v_{x}+u_{y}v_{y}+u_{z}v_{z}\\ \left|\vec{u}\right|=\sqrt{{u_{x}}^{2}+{u_{y}}^{2}+{u_{z}}^{2}}\\ \left|\vec{v}\right|=\sqrt{{v_{x}}^{2}+{v_{y}}^{2}+{v_{z}}^{2}} \end{array}$$

The angle orientation at other joints are computed analogously based on the estimated location of the markers (i.e., joints) and they are tracked temporally to describe their respective movements.

## 5. Experiments and Results

In this section, we report the different experiments conducted to evaluate the performance of the proposed method and also compare it with existing similar methods. The computational complexity of the proposed method is also analyzed, and the challenges and future research directions are also discussed in this section.

### 5.1. Performance Evaluation and Comparison

We evaluate the performance of the proposed movement analysis algorithm and compare the results with the existing techniques Hesse [39] and Khan [10]. The proposed system is implemented using C++, OpenCV, and the Kinect SDK 2.0. Initially, all the markers are printed on a standard printing paper and they are attached on the patient body region either using a piece of foam or plastic-sheet to make them more durable but still deformable. The later is important when marking joints’ locations that may bend towards the direction of the marker during the treatment. The execution of the colored marker-based solution is carried out on a machine with a 3.5 GHz dual-core processor, 8 GB RAM and 128 GB solid-state drive (SSD). In actual recording in the hospital, we used a simple notebook with Intel core i5 2.6 GHz processor and 8 GB RAM.

As the markers are located at joints, the performance of the proposed algorithm to encode the motion information is computed in terms of precision in predicting the markers (i.e., joints’ locations). We use the Average Joint Position Error (AJPE) to compute the marker detection accuracy. The joint position error (JPE) represents the error in the predicted joint position to the corresponding ground truth. It is computed as the euclidean distance between the estimated joint position and its corresponding ground truth. The JPE for all joints is computed and averaged to obtain the AJPE measure. The proposed method is evaluated on an entire test dataset, and the experiments showed that it accurately detected all the markers in almost each frame, yielding a detection rate close to 100%. The average position error was computed for each joint in all the videos and their results are presented in Table 1. The results show that our algorithm outperformed the compared methods Hesse [39] and Khan [10] in all twelve body parts. The overall average joint position error of our method is 2.7 ms, which is significantly better than Hesse [39] and Khan [10] algorithms whose AJPE is 41 and 12.7, respectively.

**Figure 5:**
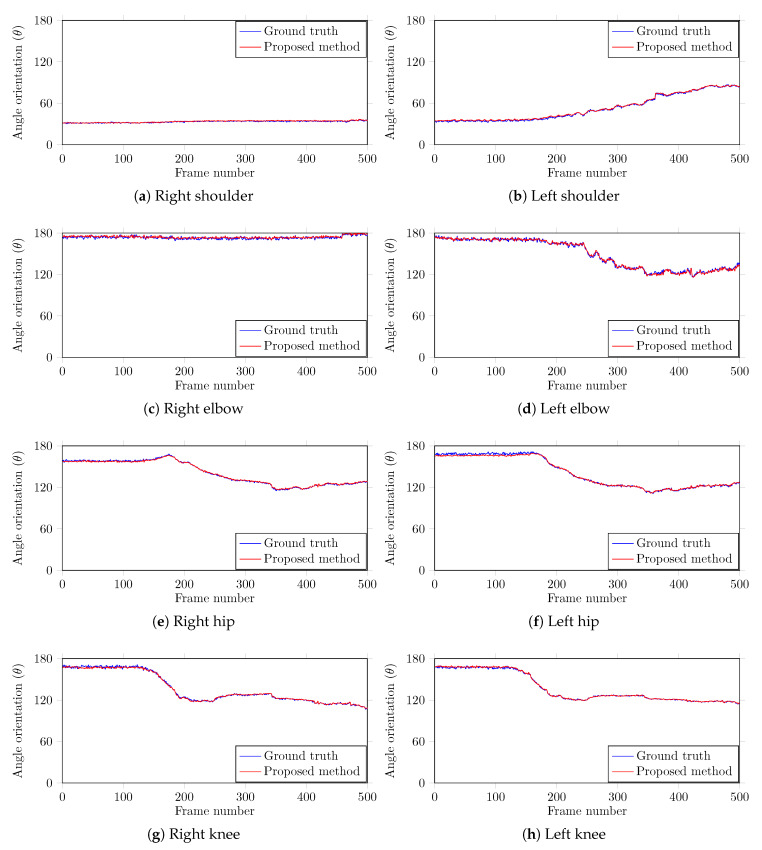
Predicted and ground truth angle orientations of different joints in a video sequence with 500 frames from our test dataset. In each graph, the angle orientation (θ) is computed in the sagittal plane.

To further investigate the performance of the proposed method, we computed the angle orientations of different body parts. Since the proposed method captures both the color and the depth frames, the angles can be computed in the 3D domain at predicted joint locations to encode their respective movement. We compare the computed angle orientations with the corresponding ground truth information that was manually recorded across all the frames to find the error in the estimated angles. A team of two members carefully analyzed each case and manually marked the positions of the joints in each test image using the center of the marker object. The body parts $Shoulder_{L}$, $Shoulder_{R}$, $Elbow_{L}$, $Elbow_{R}$, $Hip_{L}$, $Hip_{R}$, $Knee_{L}$, and $Knee_{R}$ are used in this study as they can show significant movement angles. The average error for each body part is computed and the results are outlined in Table 2. The results show that our method is highly accurate in body part angle computation with a negligible average error 0.79°. Figure 5 shows the encoded motion information of each joint and the corresponding ground truth data. The plots shows the angle estimated by our method closely follows the ground truth, demonstrating that our method is highly accurate. This movement information across time can help the doctors and the therapists to estimate the rehabilitation program based on the progress of specific motion information at a particular joint.

**Figure 6:**
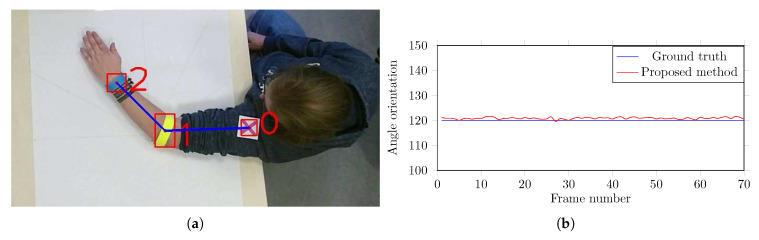
Validation of angle computation using a canvas with marked angle orientation. (**a**) The color markers are hooked-up to the arm of a proband, (**b**) the validation of computed angles at 120°.

Furthermore, in order to validate the precision of the computed angles, another experiment is conducted. Different angles were drawn onto a canvas and it was fixed on a table surface as shown in Figure 6a. The videos are captured in the same recording environment (Section 3)—the color markers are hooked-up to the arm of a proband and he is instructed to lay his arm on the canvas (Figure 6a), holding it at the exact angle given by the canvas, and one short of video sequence is recorded for each angle. Figure 6b shows the validation of computed angles at 120°. The results show that the proposed method is very accurate in computing the body part angles. The small fluctuations in computed angles is due to the unintentional movements in the proband’s arm. Since the proposed algorithm uses the center of the marker’s bounding box as the actual joint position, one has to be careful while placing the markers. If the marker is not hooked-up accurately, the angles can easily be set off by a few degrees, which may cause error in movement estimation.

From the results of the experimental evaluations presented in Table 1 and Table 2, and Figure 5 and Figure 6 reveal the efficacy of the proposed method. The system showed promising results when dealing with the occlusions. The false positives are pretty low due to outliers removal and similarity calculation. In case the system is unable to detect a marker, the proposed Kalman filtering technique with area thresholding is beneficial in estimating the marker position and preventing a notable drifting from the last detected position. The experimental evaluation of the early-stage version of the colored markers and the positive reviews from the therapists assure us that the markers do not hinder their movement and therefore were not a disruptive factor during the therapy session.

### 5.2. Computational Complexity Analysis

We also computed the computational time complexity of the proposed algorithm. The execution time on the whole dataset is computed and averaged, the results are summarized in Table 3. The results show that the proposed algorithm takes on average 59.84 ms to process one frame, leading to a performance of about 16.71 fps, which is close to real time. An efficient implementation on a better machine can further improve the time complexity of the proposed algorithm. Since Microsoft Kinect generates a huge amount of color and depth data, space requirement is an important factor to store this information. The frequent storage operation also impacts the running time of the system. In experimental evaluations, we used a system with solid state drive (SSD) to store both the RGB and depth data. The therapists need these original recordings in the starting phase of this project to manually validate the computed results of the proposed algorithm with the recorded visible movements in video. However, such recordings will not be needed in the home-based therapy setup, hence no additional storage is required for the proposed algorithm.

### 5.3. Challenges, Shortcomings, and Future Research Directions

The main challenge in the proposed system is handling the occlusion of the markers. The occlusion of the markers is unavoidable either due to the movement of the therapists or the self-occlusion of the body-parts. If a marker is occluded for a long period of time, the estimated filter’s position keeps drifting towards the direction the marker was moving in when it was last detected. This is because there is no correction stage for the Kalman filter if the marker has not been detected. Since the proposed system is designed to track human joints’ positions using markers keeping in view that the patient is lying on the table while the therapy is given, it is obvious that these positions can only move within a certain area. Therefore, the proposed algorithm measures the distance between the current position and the last detected location of the markers and prevents further tracking if this distance is greater than a certain threshold τ. In the experiments, it is observed that the estimated result was more accurate when the threshold was used. However, in the case of significant occlusions (i.e., persist for a longer period), the Kalman filtering technique may fail in estimating the marker’s position, which will drift towards the direction the marker was moving in when it was last detected. To deal with the longer occlusions, in the future, we plan to extend the proposed algorithm using multiple cameras rather than using a single camera setup. Moreover, we also intended to verify the estimation of color markers using IMU sensors.

Another future research direction of the proposed system would be to investigate its performance with a research gold standard system, for example, Motion Analysis, Vicon. Such an analysis is usually carried out in a specialized laboratory using motion capture and force plate systems [73]. These systems originally started with the analysis of gait but employed in related areas as well including VFX studios, sports therapists, neuroscientists, and in several computer vision and robotics applications. In recent studies, the Vicon (https://www.vicon.com/) motion capture system has been considered as the gold standard system to measure gait parameters with higher accuracy and reliability [74,75]. Though such solutions are considered as the gold standard method to encode the motion information and to identify alterations in joint biomechanics, they also have some disadvantages. They are quite expensive, require specialized laboratory settings, consume a lot of time during calibration and the cameras are sensitive to reflections, which makes it hard to use [76]. They are less useful for collecting data in unusual circumstances (other than walking, such as the movement of the patient in clinical settings). Hence, more efforts have been done in recent years to build an inexpensive assessment tool that can quantitatively analyze patients’ body-parts movement in clinical settings [73,76].

Though the movement of human body-parts in the proposed method is not related to walking patterns, however they are encoded using postural angles in biomechanical studies. Therefore, to confirm the reliability and accuracy of the proposed system, this angle information can be validated using a suitable measure such as mean-square error (MSE) of angular parameters between the proposed method and reference gold standard Vicon system. Since the Vicon motion capture system is quite expensive in comparison to a depth-sensing camera, inertial measurement unit, or even both of them, the movement results of the proposed system could not be compared at the moment due to financial limitations.

## 6. Conclusions

This paper presents a low-cost 3D visual tracking system to monitor the movements of the patient body-parts during the therapeutic procedure. The proposed method employs color markers with geometrical shapes, which are hooked-up at different joints and their detection in the video frames is used to estimate the joints’ locations. Later, the angles are computed at these estimated locations and their tracking in the temporal direction is used to analyze the movement of the respective joints. The performance evaluation of the proposed system was carried out on a database of 10 real patients. The experimental evaluation revealed that the proposed system is capable of encoding the patient’s body-part movement with impressive accuracy and it can be used as a rehabilitation system for in-home based therapy evaluation. In the future, we plan to extend the proposed framework using multiple cameras to record the multi-view information and motion sensors to validate the movements in the occluded regions.

## Figures and Tables

**Table 1:** Average joint position error (in millimeter) for each body part. The subscripts *R* and *L* represents the right and the left body part, respectively.

Method	${\mathrm{Shoulder}}_{R}$	${\mathrm{Shoulder}}_{L}$	${\mathrm{Elbow}}_{R}$	${\mathrm{Elbow}}_{L}$	${\mathrm{Wrist}}_{R}$	${\mathrm{Wrist}}_{L}$	${\mathrm{Hip}}_{R}$	${\mathrm{Hip}}_{L}$	${\mathrm{Knee}}_{R}$	${\mathrm{Knee}}_{L}$	${\mathrm{Ankle}}_{R}$	${\mathrm{Ankle}}_{L}$	Avg.
Hesse [39]	33.0	27.0	73.0	24.0	20.0	44.0	149.0	12.0	45.0	49.0	28.0	30.0	41.0
Khan [10]	11.9	11.0	11.4	11.2	12.4	11.9	14.4	11.2	11.9	11.7	14.0	12.8	12.7
Proposed	1.7	1.9	2.0	2.4	3.3	3.0	1.8	3.9	1.8	2.9	4.0	3.0	2.7

**Table 2:** The average error in angle estimation (in degrees) at predicted joint locations with respect to corresponding ground truth. The subscripts *R* and *L* represents the right and the left body part, respectively.

${\mathrm{Shoulder}}_{L}$	${\mathrm{Shoulder}}_{R}$	${\mathrm{Elbow}}_{L}$	${\mathrm{Elbow}}_{R}$	${\mathrm{Hip}}_{L}$	${\mathrm{Hip}}_{R}$	${\mathrm{Knee}}_{L}$	${\mathrm{Knee}}_{R}$	Average
0.63	0.58	1.10	1.16	0.85	0.65	0.64	0.71	0.79

**Table 3:** Computational complexity of the proposed algorithm.

Activities in the Proposed Algorithm	Computational Complexity
Recording of synchronized RGB and depth streams	25 fps
Average processing time per frame	59.84 ms
Number of processed frames per second	16.71 fps
